# Estimation of 16S rRNA gene copy number in several probiotic *Lactobacillus* strains isolated from the gastrointestinal tract of chicken

**DOI:** 10.1111/j.1574-6968.2008.01305.x

**Published:** 2008-08-14

**Authors:** Chin Mei Lee, Chin Chin Sieo, Norhani Abdullah, Yin Wan Ho

**Affiliations:** 1 Microbial Biotechnology Unit, Laboratory of Industrial Biotechnology, Institute of Bioscience, Universiti Putra Malaysia Selangor, Malaysia; 2 Department of Microbiology, Faculty of Biotechnology and Biomolecular Sciences, Universiti Putra Malaysia Selangor, Malaysia

**Keywords:** *Lactobacillus* spp., 16S rRNA gene copy number, rRNA restriction patterns

## Abstract

The copy numbers of 16S rRNA genes in 12 probiotic *Lactobacillus* strains of poultry origin were analyzed. Genomic DNA of the strains was digested with restriction endonucleases that do not cut within the 16S rRNA gene of the strains. This was followed by Southern hybridization with a biotinylated probe complementary to the 16S rRNA gene. The copy number of the 16S rRNA gene within a *Lactobacillus* species was found to be conserved. From the hybridization results, *Lactobacillus salivarius* I 24 was estimated to have seven copies of the 16S rRNA gene, *Lactobacillus panis* C 17 to have five copies and *Lactobacillus gallinarum* strains I 16 and I 26 four copies. The 16S rRNA gene copy numbers of *L. gallinarum* and *L. panis* reported in the present study are the first record. *Lactobacillus brevis* strains I 12, I 23, I 25, I 211, I 218 and *Lactobacillus reuteri* strains C 1, C 10, C 16 were estimated to have at least four copies of the 16S rRNA gene. In addition, distinct rRNA restriction patterns which could discriminate the strains of *L. reuteri* and *L. gallinarum* were also detected. Information on 16S rRNA gene copy number is important for physiological, evolutionary and population studies of the bacteria.

## Introduction

Lactobacilli, the predominant species in the avian alimentary tract, are Gram-positive, nonsporing, catalase-negative, devoid of cytochromes and acid tolerant bacteria. They are strictly fermentative, with lactic acid as the major end-product during carbohydrate fermentation. The concept of using lactobacilli as probiotics to exert health benefits has drawn the attention of the public in recent years, particularly following the total ban or severe restriction of antibiotics as growth promoters in the animal industry. With the growing interest in the use of probiotics, it is essential to have a rapid and accurate method for assessing probiotic preparations. Various molecular techniques, which are known to be rapid, accurate and sensitive, could be employed for this purpose. The most widely used techniques for bacterial identification and quantification are based on the 16S rRNA gene. Previous studies have indicated that the 16S rRNA gene is present in multiple copies in most bacterial genomes and the copy numbers vary from one to as many as 15 (Acinas *et al.*, 2004). The 16S rRNA gene copy number is useful for 16S rRNA gene-based quantification technique. Data obtained from samples for the estimation of microbial community should be normalized with the 16S rRNA gene copy number per cell to avoid quantitative bias.

To date, *Lactobacillus* 16S rRNA gene copy number has only been characterized in a few species. *Lactobacillus acidophilus* has been reported to comprise four copies of the 16S rRNA gene (Roussel *et al.*, 1993; Altermann *et al.*, 2005). *Lactobacillus plantarum* (Chevallier *et al.*, 1994; Kleerebezem *et al.*, 2003) and *Lactobacillus brevis* (Makarova *et al.*, 2006) contain five copies. Six copies of the 16S rRNA gene are found in the genome of *Lactobacillus johnsonii* (Pridmore *et al.*, 2004), *Lactobacillus gaseri* (Makarova *et al.*, 2006) and *Lactobacillus reuteri* (GenBank accession number CP000705). *Lactobacillus sakei* (Dudez *et al.*, 2002) and *Lactobacillus salivarius* (Claesson *et al.*, 2006) possess seven copies of the 16S rRNA gene per genome, while *Lactobacillus delbrueckii* has the highest multiplicity, with nine copies of the 16S rRNA gene (Makarova *et al.*, 2006; van de Guchte *et al.*, 2006). Information on other *Lactobacillus* species is scarce. Thus, in the present study, the 16S rRNA gene copy numbers of 12 *Lactobacillus* strains from five different species (*Lactobacillus gallinarum*, *Lactobacillus panis*, *L. reuteri*, *L. brevis* and *L. salivarius*) were determined by Southern hybridization technique. Seven restriction enzymes that do not cut within the 16S rRNA genes were used to digest the genomic DNA of the strains. The digested genomic DNA was hybridized with a labeled probe which was complementary to the 16S rRNA gene to determine the 16S rRNA gene copy number.

## Materials and methods

### Bacterial strains

*Lactobacillus reuteri* C 1, C 10 and C 16; *L. brevis* I 12, I 23, I 25, I 211 and I 218; *L. gallinarum* I 16 and I 26; *L. salivarius* I 24; and *L. panis* C 17 used in this study were previously isolated from the gastrointestinal tract of chickens by Jin *et al.* (1996). These *Lactobacillus* strains were identified using conventional and molecular techniques, and were used as a probiotic to exert health benefits in poultry (Jin *et al.*, 1998, 2000; Kalavathy *et al.*, 2003; Lee *et al.*, 2008). The *Lactobacillus* strains were propagated in de Man, Rogosa, Sharpe (MRS) broth (Oxoid, UK) and incubated at 39 °C for 24 h in anaerobic jars (Oxoid) with gas generating kits (Oxoid). Stock cultures were stored in 15% glycerol at −80 °C.

### Genomic DNA preparation

High-molecular-weight DNA was extracted from the 12 *Lactobacillus* strains according to the method described by Leenhouts *et al.* (1990) with slight modifications as follows. The overnight bacterial cultures (24 h) were resuspended in lysis buffer containing 8 mg of lysozyme mL^−1^ and incubated at 37 °C for 2 h. After 1-h incubation with proteinase K and sodium dodecyl sulphate (SDS), the lysate was extracted twice with an equal volume of phenol–chloroform–isoamyl alcohol (25 : 24 : 1) and once with an equal volume of chloroform–isoamyl alcohol (24 : 1). Genomic DNA was dissolved in 50 μL of TE buffer after precipitation with 3 M sodium acetate and ethanol.

### Restriction enzyme digestion of DNA and agarose gel electrophoresis

The 16S rRNA gene sequences of the 12 *Lactobacillus* strains were analyzed using NEB cutter (Vincze *et al.*, 2003) to determine the enzymes that do not cut within the 16S rRNA gene of the strains (GenBank accession numbers EF412975–EF412986). Genomic DNA (10 μg) was digested with a range of noncutting restriction enzymes (BamHI, BclI, EcoRI, EcoRV, HindIII, NheI and SalI; New England BioLabs). Independent digestions were carried out with 20 units of restriction enzyme and incubated overnight at their optimum conditions as recommended by the manufacturer. Digested DNA was subjected to electrophoresis on a 0.8% (w/v) agarose gel in Tris-acetate buffer at 60 V for 4 h. DNA fragments were transferred to Amersham Hybond N+(GE Healthcare) by capillary blotting (Sambrook *et al.*, 1989) with 20 × SSC as the transfer solution. After the transfer, the nylon membrane was dried and cross-linked by UV irradiation.

### Preparation of biotinylated 16S rRNA gene probe

The probe was prepared by PCR amplification of a fragment of the 16S rRNA gene from *L. reuteri* C 10 which corresponded to the conserved sequence complementary to the positions 8-519 region of the rRNA gene (*Escherichia coli* numbering; Brosius *et al.*, 1978). PCR mixture contained 50–100 ng of bacterial DNA, 50 pmol of each primer, 10 μmol of dNTPs (Finnzyme, Finland), 1 U of DyNAzyme II DNA polymerase (Finnzyme), and 1 × PCR buffer, and the volume was adjusted to 50 μL with deionized water. PCR amplification with universal primers 8f (5′-AGAGTTTGATCCTGGCTCAG-3′) and 519r (5′-GTATTACCGCGGCTGCTGG-3′) was carried out by an initial denaturation at 95 °C for 5 min, 30 cycles of 95 °C for 1 min, 55 °C for 1 min, 72 °C for 2 min and final extension at 72 °C for 7 min. The PCR products were electrophoresed and purified using the Qiaquick PCR purification kit (Qiagen, Germany). The purified products were biotinylated by random labeling with the NEBlot Phototope Kit (New England BioLabs) according to the manufacturer's instructions.

### Southern hybridization analysis

Before hybridization, prehybridization was carried out for 1 h at 60 °C in 5 × Denhardt's solution, 6 × SSC, 0.5% (w/v) SDS, and 10 μg mL^−1^ denatured salmon sperm DNA. Hybridization was accomplished by the addition of 20 ng mL^−1^ of denatured biotinylated probe to the same solution and incubated at 60 °C overnight. After hybridization, the blots were washed twice with 2 × SSC containing 0.1% (w/v) SDS at room temperature for 5 min each and twice with 0.5 × SSC containing 0.1% (w/v) SDS for 15 min each at 60 °C. Detection of hybridized probe was performed using a chemiluminescent detection system (Phototope-Star Detection Kit, New England BioLabs). Hybridized DNA was visualized by exposure of the membrane to X-ray film (Kodak).

## Results and discussion

The estimation of 16S rRNA gene copy number is very much dependent on the restriction digestion of genomic DNA by appropriate restriction endonucleases. Over or underestimation of copy numbers may occur. Although overestimation of the copy numbers of 16S rRNA genes may be relatively rare, it could happen if the target sequence of the 16S rRNA gene probe is cleaved by the restriction enzyme (Maslunka *et al.*, 2006). Thus, in the present study, sequence analysis of the 16S rRNA gene was performed using NEB cutter (Vincze *et al.*, 2003) to select restriction enzymes that do not cleave within the 16S rRNA gene, for the estimation of the gene copy number. A poor electrophoretic separation of DNA fragments is one of the factors that results in the underestimation of 16S rRNA gene copy numbers. Large DNA fragment or two DNA fragments of very similar size are always difficult to separate and this will result in a single hybridization fragment containing two or more 16S rRNA genes. Besides, large DNA fragments which contain more than one gene with no cutting site between them will also contribute to the underestimation of 16S rRNA gene copy numbers. Performing several independent restriction digests is, therefore, essential in order to obtain an accurate estimation of 16S rRNA gene copy number of a bacterium. In the present study, different digestions of genomic DNA that produced similar number of hybridizing bands were used to determine the copy numbers of the 16S rRNA gene and, for each digest, a distinct hybridizing band detected on the blot corresponded to at least one 16S rRNA gene copy per genome.

**Fig. 1:**
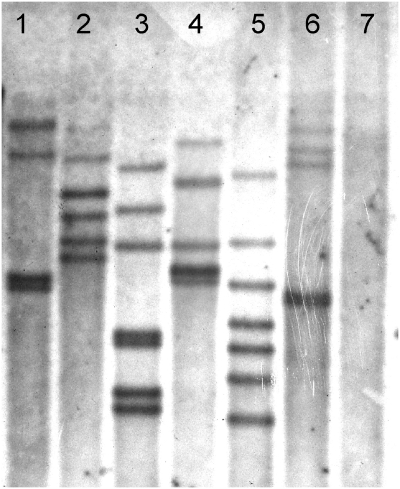
16S rRNA gene copy number of *Lactobacillus salivarius* I 24 as determined by Southern hybridization with biotinylated 16S rRNA gene probe. Genomic DNA of *L. salivarius* I 24 was cleaved with several noncutting restriction enzymes. Lane 1, BamHI; lane 2, BclI; lane 3, EcoRI; lane 4, EcoRV; lane 5, HindIII; lane 6, NheI; lane 7, SalI.

The results of Southern hybridization of *L. salivarius* I 24 genomic DNA with the probe complementary to the 16S rRNA gene revealed that the hybridization of EcoRI and HindIII fragments yielded seven bands (Fig. 1), which indicated that *L. salivarius* I 24 probably possessed seven copies of the 16S rRNA gene in the genome. This result is in agreement with the recent finding of Claesson *et al.* (2006) in which the complete genome sequence analysis of *L. salivarius* strain UCC 118 shows that the strain has seven copies of the 16S rRNA gene.

**Fig. 2:**
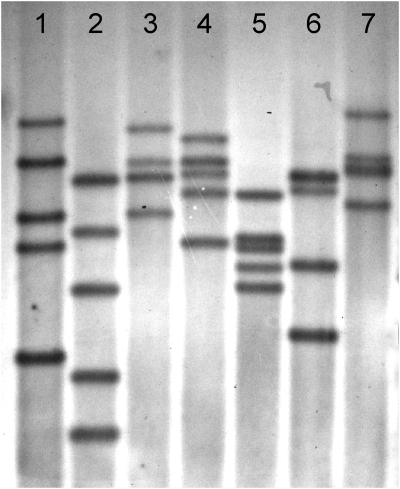
Southern hybridization of *Lactobacillus panis* C 17 genomic DNA digested with different restriction enzymes. Lane 1, BamHI; lane 2, BclI; lane 3, EcoRI; lane 4, EcoRV; lane 5, HindIII; lane 6, NheI; lane 7, SalI.

**Fig. 3:**
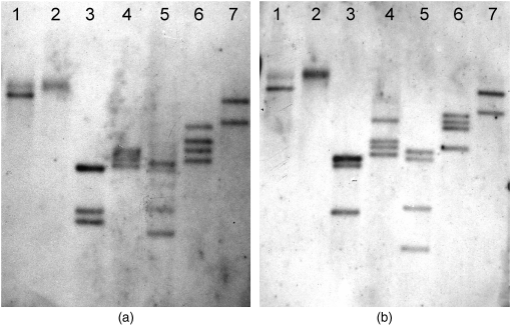
Southern hybridization with genomic DNA of *Lactobacillus gallinarum* I 16 (a) and I 26 (b). The genomic DNA was cleaved with several restriction enzymes that do not cut within the 16S rRNA gene. Lane 1, BamHI; lane 2, BclI; lane 3, EcoRI; lane 4, EcoRV; lane 5, HindIII; lane 6, NheI; lane 7, SalI.

There has been no information on the 16S rRNA gene copy numbers of *L. gallinarum* and *L. panis*. In the present study, the 16S rRNA gene probe hybridized to five bands in the BamHI, BclI, EcoRV and HindIII digests for *L. panis* C 17 (Fig. 2). Thus, the 16S rRNA gene copy number was estimated to be five. As for *L. gallinarum* I 16 and I 26, four 16S rRNA gene copy numbers were estimated from EcoRV, HindIII, and NheI digests (Fig. 3). However, unlike the gene copy number, the hybridization patterns of these digests were not similar between the two *L. gallinarum* strains.

**Fig. 4:**
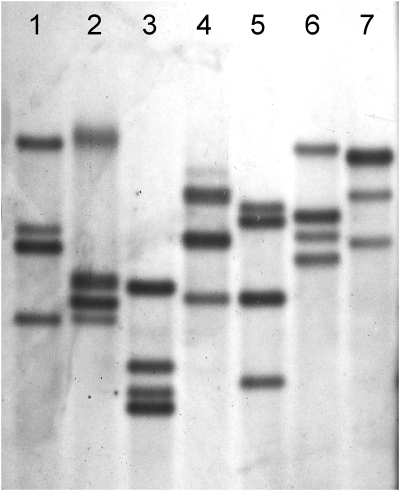
Southern hybridization of *Lactobacillus brevis* I 211 genomic DNA digested with different restriction enzymes. Lane 1, BamHI; lane 2, BclI; lane 3, EcoRI; lane 4, EcoRV; lane 5, HindIII; lane 6, NheI; lane 7, SalI.

When Southern blots of *L. brevis* strains I 12, I 23, I 25, I 211 and I 218 were hybridized with the 16S rRNA gene probe, uniform hybridization patterns were obtained for the five *Lactobacillus* strains (representative gel in Fig. 4). Similar hybridization patterns and number of hybridizing bands were produced for all five strains when their chromosomal DNA was digested with BamHI, BclI, EcoRI, HindIII and NheI. The results of the Southern blots revealed the presence of four hybridizing bands of varying intensities, which suggests the existence of at least four copies of 16S rRNA gene in the *L. brevis* strains. As some of the bands have much stronger signals, it may suggest the presence of two or more comigrating DNA fragments or two or more 16S rRNA genes located on the same DNA fragment. Recently, Makarova *et al.* (2006) reported that based on the complete genome sequences of *L. brevis* ATCC 367 in the GeneBank, the strain possessed five copies of the 16S rRNA genes.

**Fig. 5:**
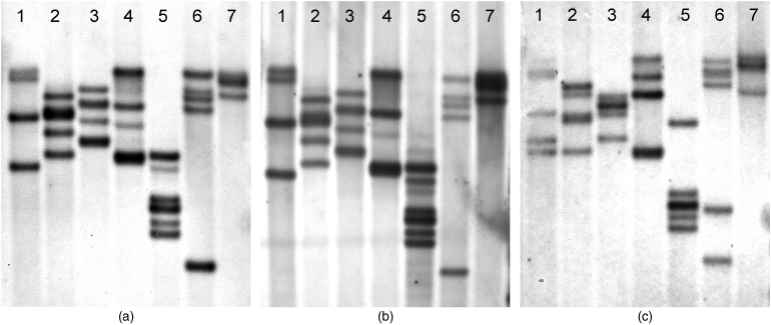
Southern blot analysis of the 16S rRNA gene of *Lactobacillus reuteri* C 1 (a), C 16 (b) and C 10 (c). Several enzymes were used to digest genomic DNA of the *Lactobacillus* strains. Lane 1, BamHI; lane 2, BclI; lane 3, EcoRI; lane 4, EcoRV; lane 5, HindIII; lane 6, NheI; lane 7, SalI.

Digestion of chromosomal DNA of *L. reuteri* C 1, C 10 and C 16 with BamHI, BclI, EcoRI and EcoRV yielded four hybridizing bands of varying intensities, indicating the existence of at least four copies of the 16S rRNA gene (Fig. 5). It was also observed that the hybridizing patterns of these digests and those of HindIII, NheI and SalI for *L. reuteri* C 1 and C 16 were identical (Fig. 5a and b). The full genome sequencing of *L. reuteri* DSM 20016^T^ (GenBank accession number CP000705) was completed recently and it was found that the 16S rRNA gene was present in six copies. The discrepancy between this result and that of the present study suggests that the hybridizing bands with strong signals in the present study could contain more than one 16S rRNA gene.

The 16S rRNA gene copy numbers showed relatively little variation among strains within a species. As pointed out by Johansen *et al.* (1996), variations in 16S rRNA gene copy numbers between different bacterial species are well documented, but strain-dependent differences are reported at a lower rate. In contrast to the 16S rRNA gene copy number, the restriction fragment patterns on Southern blots can be diverse among the strains. The variations of the hybridizing patterns may be due to genomic heterogeneity caused by point mutations, genetic rearrangements and additive genetic changes within and between the gene (Liu & Sanderson, 1998; Acinas *et al.*, 2004; González-Escalona *et al.*, 2005). These events may change the cutting site frequency of restriction enzyme and the size of restricted fragment which subsequently generate substantial differences in the hybridization patterns. Thus, 16S rRNA gene restriction patterns seem to represent a promising tool in typing bacterial strains. Hybridization patterns of the 16S rRNA gene have been successfully used to discriminate strains of some lactic acid bacteria (Björkroth & Korkeala, 1997) and *Lactobacillus* strains (Rodtong & Tannock, 1993; Björkroth & Korkeala, 1996; Simpson *et al.*, 2001). Similarly, in the present study, *L. reuteri* C 10 could be differentiated from *L. reuteri* C 1 and C 16, and *L. gallinarum* I 16 from *L. gallinarum* I 26 based on the hybridization patterns of these strains.

Although many studies have been carried out on the multiplicity of the 16S rRNA gene in prokaryotic organisms, little information is available for *Lactobacillus* species. To our knowledge, no data on the 16S rRNA gene copy numbers of *L. gallinarum* and *L. panis* have been reported and our study is the first record. Information on 16S rRNA gene multiplicity is potentially useful in physiological and evolutionary study of bacteria. In addition, copy numbers of the 16S rRNA gene are important as a correction factor to normalize the data collected on the estimation of a bacterial community, especially when technology such as quantitative real-time PCR is employed.

## Statement

Reuse of this article is permitted in accordance with the Creative Commons Deed, Attribution 2.5, which does not permit commercial exploitation.
